# Depletion of ATP and glucose in advanced human atherosclerotic plaques

**DOI:** 10.1371/journal.pone.0178877

**Published:** 2017-06-01

**Authors:** Matias Ekstrand, Emma Widell, Anna Hammar, Levent M. Akyürek, Martin Johansson, Björn Fagerberg, Göran Bergström, Malin C. Levin, Per Fogelstrand, Jan Borén, Max Levin

**Affiliations:** 1 Department of Molecular and Clinical Medicine, Wallenberg Laboratory, University of Gothenburg and Sahlgrenska University Hospital, Gothenburg, Sweden; 2 Department of Pathology, Sahlgrenska University Hospital, Gothenburg, Sweden; 3 Department of Pathology, Malmö University Hospital, Lund University, Malmö, Sweden; 4 Department of Oncology, Sahlgrenska University Hospital, Gothenburg, Sweden; Monash University, AUSTRALIA

## Abstract

**Objective:**

Severe hypoxia develops close to the necrotic core of advanced human atherosclerotic plaques, but the energy metabolic consequences of this hypoxia are not known. In animal models, plaque hypoxia is also associated with depletion of glucose and ATP. ATP depletion may impair healing of plaques and promote necrotic core expansion. To investigate if ATP depletion is present in human plaques, we analyzed the distribution of energy metabolites (ATP, glucose, glycogen and lactate) in intermediate and advanced human plaques.

**Approach and results:**

Snap frozen carotid endarterectomies from 6 symptomatic patients were analyzed. Each endarterectomy included a large plaque ranging from the common carotid artery (CCA) to the internal carotid artery (ICA). ATP, glucose, and glycogen concentrations were lower in advanced (ICA) compared to intermediate plaques (CCA), whereas lactate concentrations were higher. The lowest concentrations of ATP, glucose and glycogen were detected in the perinecrotic zone of advanced plaques.

**Conclusions:**

Our study demonstrates severe ATP depletion and glucose deficiency in the perinecrotic zone of human advanced atherosclerotic plaques. ATP depletion may impair healing of plaques and promote disease progression.

## Introduction

Cells need oxygen and nutrients to produce ATP required for ion pumps, migration and intracellular metabolism. In the artery wall, oxygen and nutrients are supplied via diffusion from luminal blood and vasa vasorum [1, 2]. In atherosclerosis-prone large and medium-sized arteries, diffusion distances are long. During atherogenesis, diffusion distances increase further as the intima grows thicker. At the same time, the energy metabolic demand increase due to accumulation of macrophage foam cells with high oxygen and glucose consumption [3]. As a consequence, lack of oxygen and nutrients may develop deep in plaques thus promoting energy (ATP) depletion.

ATP depletion within atherosclerotic plaques may promote progression of atherosclerosis [4]. In support of the ATP depletion hypothesis, hypoxic zones exist in human and animal plaques [5, 6]. However, the energy metabolic consequences of hypoxia in human plaques have been poorly investigated. Hypoxia is mainly detected in macrophage-rich areas deep in advanced plaques, adjacent to the necrotic core. However, macrophages adapt to hypoxia by increased anaerobic glycolysis [6–9]. Consequently, macrophages can maintain ATP levels in hypoxic microenvironments as long as glucose is available [7]. However, experimental data from animal studies show that also glucose and ATP is depleted in the macrophage-rich core of advanced plaques [8].

In the current study, we mapped concentrations of ATP, glucose, glycogen and lactate within human atherosclerotic plaques. Our results demonstrate severe cellular ATP depletion and lack of glucose in advanced human plaques, in particular close to the necrotic core. These results support the ATP depletion hypothesis.

## Materials and methods

### Study design

Energy metabolites were measured in human plaques from carotid endarterectomies. The endarterectomies were obtained from the Gothenburg Atheroma Study Group biobank. The patients had a high grade symptomatic stenosis (≥70% stenosis of the carotid artery) and underwent carotid endarterectomy at the Sahlgrenska University Hospital (Gothenburg, Sweden) during 2005 or 2006. Patient characteristics and time from last symptom until surgery are shown in Table 1. The research has been carried out in accordance with the Declaration of Helsinki of the World Medical Association. Study approval was obtained from the Gothenburg Regional Ethics Committee, and all patients gave written informed consent to participate.

**Table 1:** Patient characteristics (n = 6).

Patient characteristics^a^	
Age, years	64.4 (58–81)
*Gender*	
Male	4 (67%)
Female	2 (33%)
*Type of clinical event*	
Amaurosis fugax	3 (50%)
Stroke	3 (50%)
Time from last clinical event to surgery (days)	22.8 (9–40)
*Cardiovascular risk factors*	
Diabetes	5 (50%)
Hypertension	5 (83%)
Smoking	1 (17%)
*Blood chemistry concentrations*^b^	
HDL, mmol/L	0.88 ± 0.29
LDL, mmol/L	2.21 ± 0.74
*Medication*	
Statins	6 (100%)
Diuretics	2 (33%)
ACE-I or ARB	3 (50%)
Beta blocker	2 (33%)
Calcium antagonist	3 (50%)
Anti-platelet	5 (83%)
Oral anti-diabetics	3 (50%)
Insulin	2 (33%)

ACE-I = angiotensin converting enzyme inhibitor

ARB = angiotensin receptor blocker

HDL = high-density lipoprotein

LDL = low-density lipoprotein

^a^ Continuous data are presented as mean (range low-high) or mean (± SD) and categorical data as number (%).

^b^ n = 4; information on blood samples could not be obtained for all patients.

It is known from previous studies that plaque severity (AHA type) varies within carotid plaques. Typically, plaque histology is more advanced upstream of the carotid bifurcation, in the internal carotid artery (ICA), than in downstream portions of the same plaque, in the common carotid artery (CCA) [10]. To obtain plaques with different severity from each patient, we therefore analyzed one level in the ICA upstream of the carotid bifurcation and one level in the CCA downstream of the bifurcation. To ensure simultaneous freezing of ICA and CCA, we only analyzed endarterectomies that had been surgically removed in one intact piece (n = 6).

### AHA classification

Plaque histology was analyzed by Mayer’s HTX and eosin. Frozen 8 μm sections were incubated in Mayer’s HTX (Histolab Products AB) for 90 seconds and in eosin for 60 seconds, after which they were dehydrated in ethanol and mounted with Pertex (Histolab Products AB). Each H&E-stained section was classified by a pathologist (LMA) using AHA classification [11].

### Histology and immunofluorescence

Macrophage and smooth muscle cell content was analyzed by immunofluorescence staining of acetone fixed frozen 8 μm sections. Macrophages were labeled with an anti-CD68 antibody (1:50, mouse anti human CD68, M0718, Dako). Secondary detection was made using donkey Fab fragment anti mouse 488 (1:250, 715-547-003, Jackson Immunoresearch) after which a directly conjugated anti-α-actin antibody (mouse, conjugated to Cy3, C6198, Sigma Aldrich Co. LLC.) was added. Isotype controls were made using mouse IgG1 (X0931, Dako). Cell nuclei were stained using DAPI. Slides were scanned using a Metafer Slide Scanning Platform (MetaSystems GmbH) with a 10x objective. Quantification of CD68, α-actin, necrotic core area, cellularity, and intimal thickness was performed in ImageJ (version 1.50c, National Institutes of Health, USA).

Neutral lipid accumulation was assessed by staining one cryosection (8 μm) of each sample with Oil red O. Sections were fixed in 2% paraformaldehyde in PBS during 4 minutes, rinsed in tap water, treated with 20% isopropanol in water for 30 seconds, after which oil red O working solution was added during 7 minutes. The sections were treated with 20% isopropanol in water during 30 seconds, and rinsed in tap water for two minutes. The slides were treated with Mayers HTX (Histolab Products AB) during 1 minute, rinsed in tap water, distilled water, and finally mounted with Mowiol.

### Quantification of energy metabolites

ATP, glucose, glycogen and lactate was measured by bioluminescence imaging according to previously published protocols [12]. Bioluminescence imaging is an in vitro method, but analysis of snap frozen tissue allows assessment of the in vivo situation. To avoid enzyme degradation of metabolites, frozen tissue sections are immediately heat inactivated after sectioning (95°C for 5 minutes). Bioluminescence imaging utilizes an enzyme solution to selectively break down the desired substrate in a frozen tissue section to create a substrate for a bioluminescent enzyme [1, 12]. The photons generated by the bioluminescent enzyme are registered by a highly sensitive photon-counting camera connected to a microscope. In the resulting photon counting image, the grey value is dependent on the local concentration of the metabolite. To calibrate the bioluminescence signal, standard sections with known content of metabolites were used. Standard curves were then used to transform bioluminescence intensity to absolute concentrations. Plaque concentrations of extracellular metabolites (glucose and lactate) are expressed as millimolar (mM) whereas plaque concentrations of intracellular metabolites (ATP and glycogen) were normalized to the number of cells in the plaque. ATP is present both within and outside cells, but the intracellular concentration is 10^3^ to 10^6^ times higher than the extracellular concentration. Therefore, ATP assessment almost exclusively reflects intracellular ATP.

### Statistics

**Fig 1:**
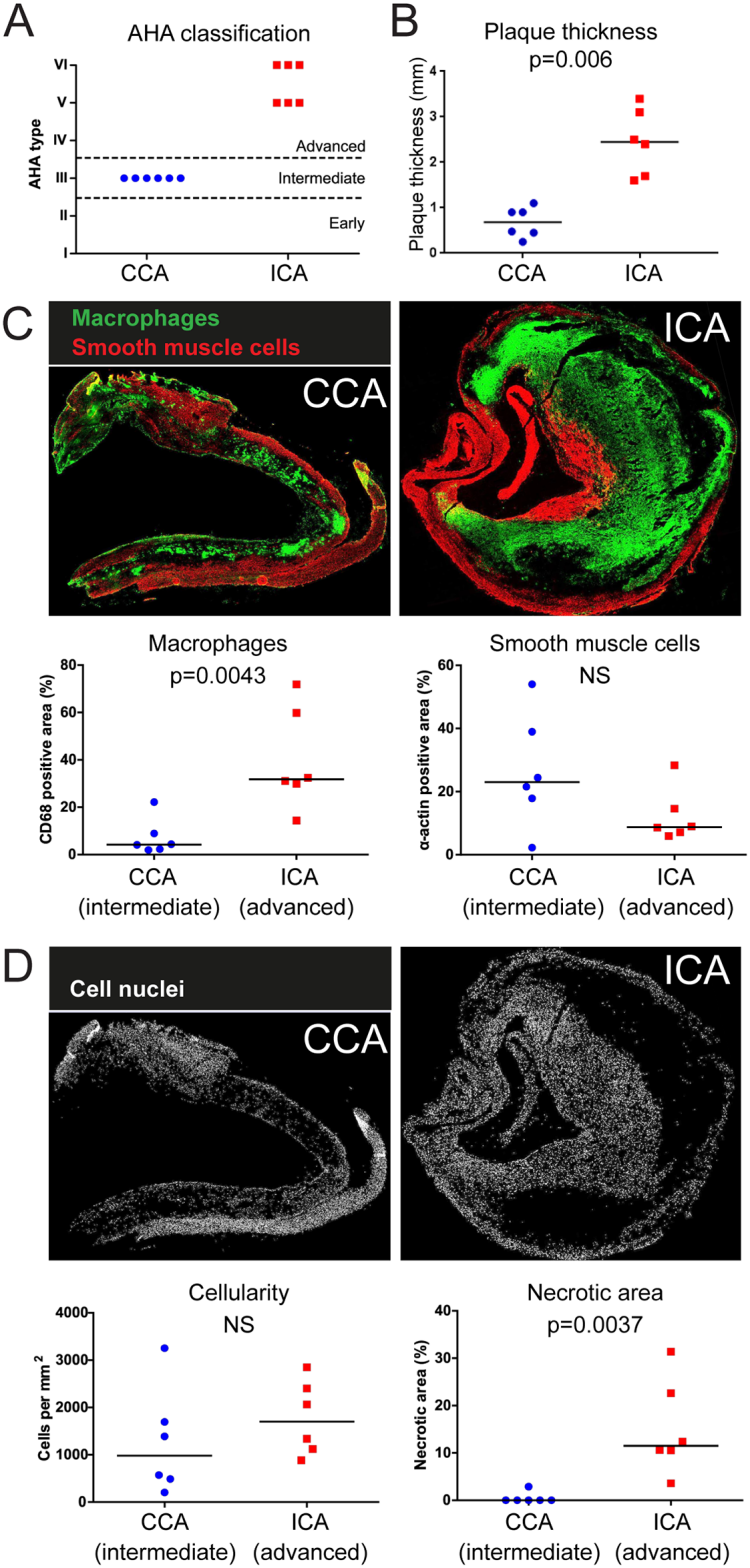
Plaques were intermediate in common carotid artery (CCA) and advanced in internal carotid artery (ICA). A. Plaques were obtained from CCA and ICA of human endarterectomies. Plaques in CCA were intermediate and plaques in ICA were advanced. B, C and D. Advanced plaques where thicker (B) and had higher macrophage content than intermediate plaques (C). There was no significant difference in smooth muscle cell content (C) or cellularity (D). Necrotic areas were larger in advanced plaques (D). n = 6, t-test (B) or Wilcoxon Signed-Rank Test (C and D).

**Fig 2:**
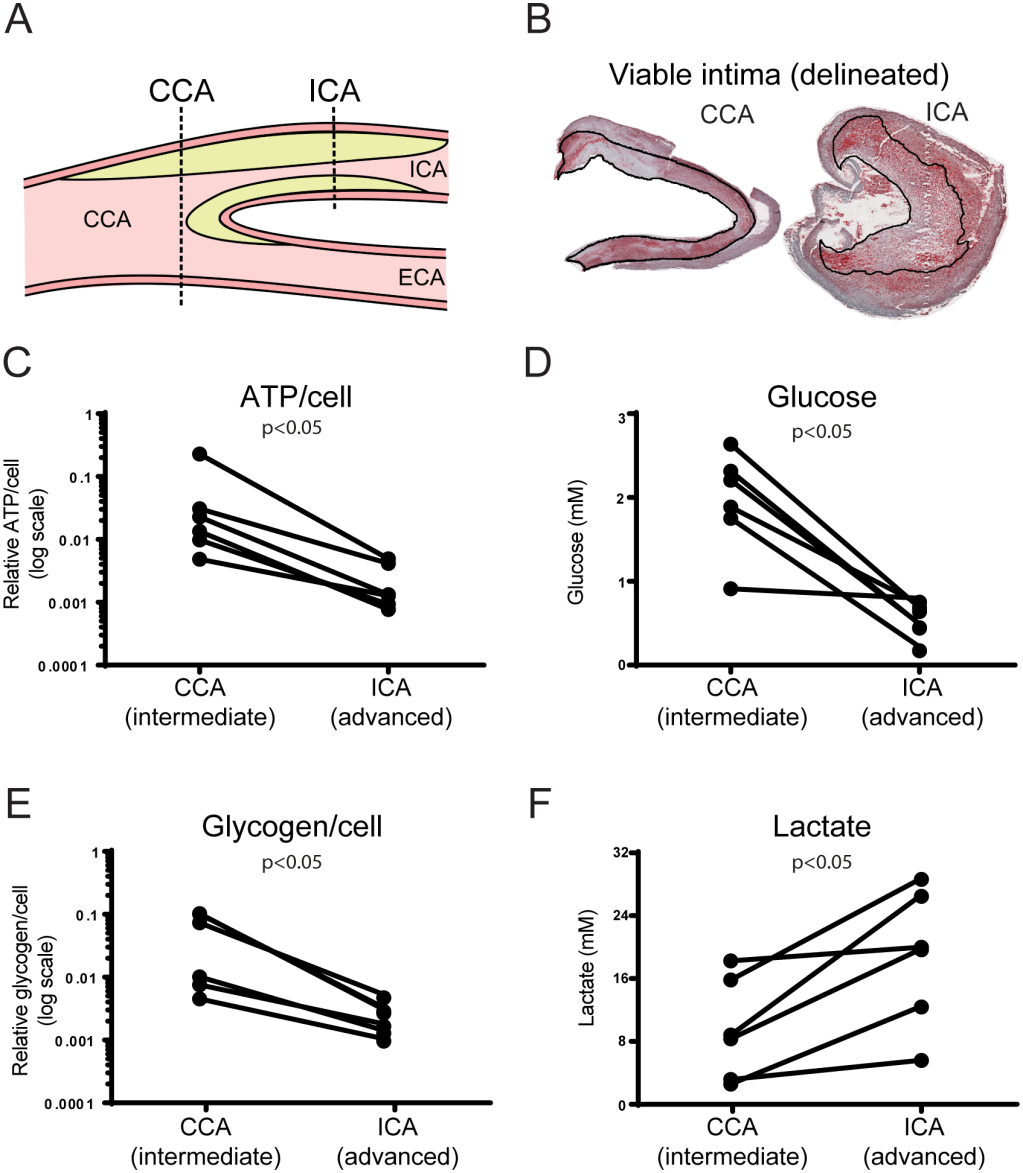
Depletion of ATP, glucose and glycogen in advanced human plaques. A. Energy metabolites were analyzed in intermediate (CCA) and advanced (ICA) segments of human endarterectomies. B. Metabolite concentrations were assessed in the viable part of the intima (delineated), i.e. intimal area minus necrotic core. C, D and E. ATP (C), glucose (D) and glycogen concentrations (E) were lower in advanced segments than in intermediate segments of the same plaque. Note logarithmic scale for ATP and glycogen. F. Lactate concentrations were higher in advanced segments of the plaque. n = 6, Wilcoxon Signed-Rank Test.

**Fig 3:**
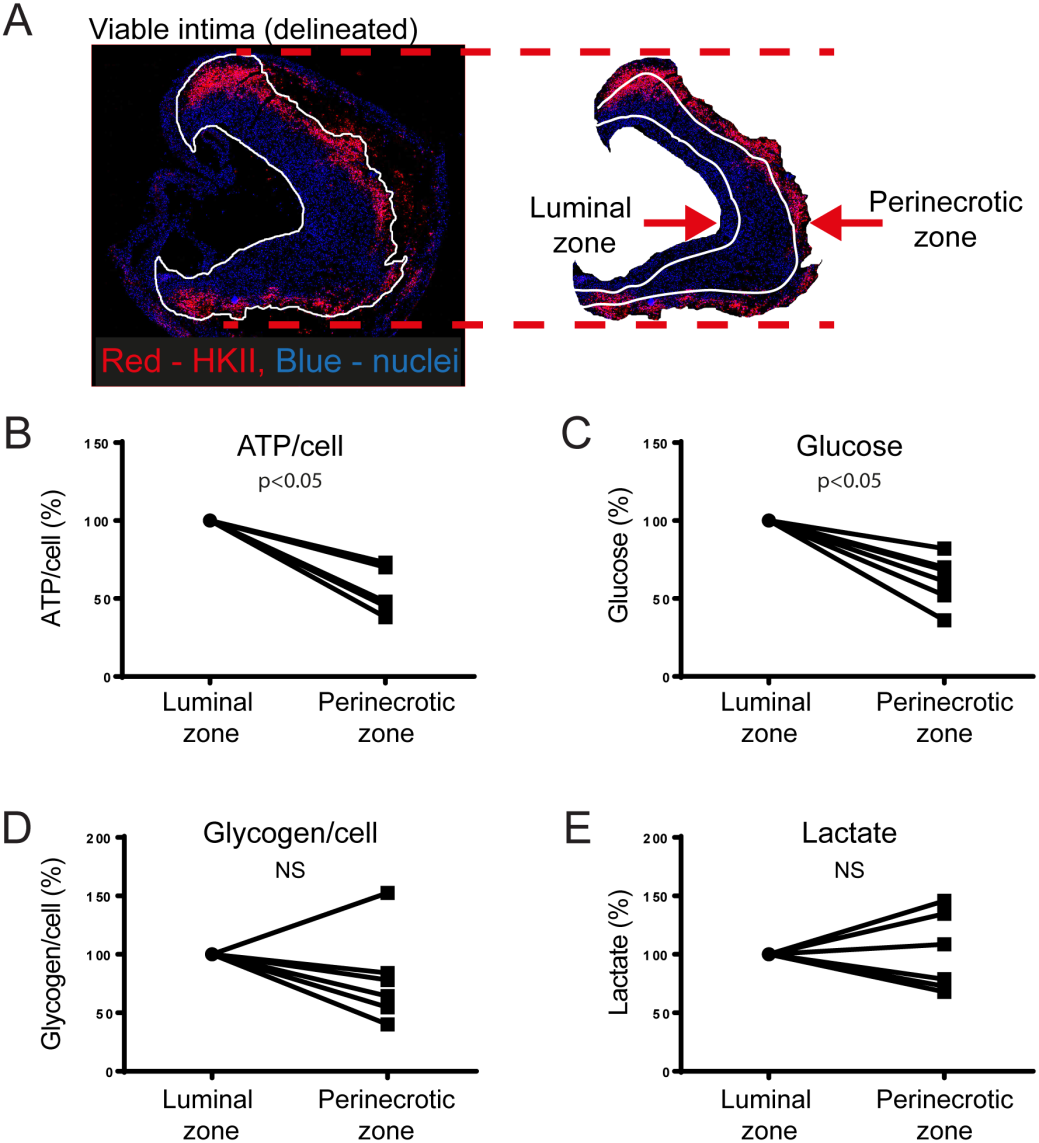
Severe depletion of ATP and glucose in perinecrotic zone of advanced plaques. A. Advanced atherosclerotic plaque, delineation in white of viable intima. Extract shows luminal (left) and perinecrotic zone (right) of viable intima. Note high expression of hexokinase II (HKII), indicative of hypoxia, in perinecrotic zone. B and C. Lower concentrations of ATP (B) and glucose (C) in perinecrotic zone (p<0.05). D and E. No significant difference in glycogen (D) and lactate concentrations (E) between luminal and perinecrotic zone. n = 6, paired t-test.

Data are expressed with median values in all graphs. p values were calculated using unpaired t-test (Fig 1B), Wilcoxon Signed-Rank Test (Figs 1C, 1D and 2) or paired t-test (Fig 3). GraphPad Prism software (v. 6.04) was used for statistical analysis.

## Results

To investigate energy metabolism in human atherosclerotic plaques, we analyzed snap frozen human carotid endarterectomies. Each endarterectomy contained a large plaque ranging from the common carotid artery (CCA) to the internal carotid artery (ICA). Plaque severity varied within each plaque and plaques in the common carotid artery were intermediate (Type III, n = 6) whereas plaques in the internal carotid artery were advanced (Type V, n = 3 or VI, n = 3) (Fig 1A), in agreement with previous observations [10]. Advanced plaques where thicker (Fig 1B), had significantly higher content of macrophages (Fig 1B) and more extensive necrotic areas (Fig 1C). Cellularity and smooth muscle cell content was not significantly different between intermediate and advanced plaques (Fig 1B and 1C). Since each plaque contained both an intermediate and advanced segment, pairwise comparisons could be made.

First, we investigated if there was a difference in intimal concentrations of energy metabolites between intermediate and advanced segments of carotid plaques (Fig 2C–2F). The mean ATP concentration per cell in advanced portions (ICA) of the plaque was only 10% (10% ± 8%; n = 6; p<0.05) of the mean ATP concentration in intermediate portions (CCA) of the same plaque (Fig 2C). Similarly, both extracellular glucose concentrations (34% ± 27%; n = 6; p<0.05) and intracellular glycogen (12% ± 9%; n = 6; p<0.05) were much lower in advanced plaques (ICA) (Fig 2D and 2E). Taken together, our results demonstrate depletion of both nutrients (glucose, glycogen) and ATP in advanced plaques. In agreement, lactate concentrations were higher in advanced plaques (248% ± 132%, n = 6; p<0.05) indicative of higher rate of glycolysis (Fig 2F).

We then investigated how energy metabolites are distributed within advanced plaques. Hypoxia in advanced plaques was mainly present in the perinecrotic zone, as indicated by high expression of hexokinase II; a glycolytic enzyme upregulated by hypoxia (Fig 3A) [13]. This agrees with previous observations [5, 6]. Interestingly, ATP (53% ± 14%, n = 6, p<0.01) and glucose (61% ± 16%, n = 6, p<0.01) concentrations were lower in the hypoxic perinecrotic zone than in the luminal zone (Fig 3B and 3C). There was no significant difference in glycogen (79% ± 40%, n = 6, non-significant) or lactate (101% ± 33%, n = 6, non-significant) concentrations between the two zones (Fig 3D and 3E).

## Discussion

In this study, we demonstrate depletion of energy metabolites in advanced human atherosclerotic plaques. The most pronounced energy depletion was detected adjacent to the necrotic core.

ATP was severely depleted in advanced plaques, in particular in the perinecrotic zone adjacent to the necrotic core. Accumulation of dead macrophages in the necrotic core promotes lesion instability and is a key event in atherosclerosis progression. Macrophages in plaques die either via apoptosis or necrosis [14]. Apoptosis is an active process requiring ATP whereas necrosis is the result of severe insults such as ATP depletion or high intracellular calcium levels. Our data demonstrating severe ATP depletion in macrophages in the perinecrotic zone suggests that necrosis may be important in this zone. This agrees with morphological studies showing that necrosis is the dominating form of cell death in human plaques [14].

ATP depletion may also impair lipid removal from plaques. Macrophage foam cells in plaques need ATP to transfer intracellular lipids to HDL particles and subsequent clearance via lymph vessels [15]. Macrophages also need ATP to remove dead lipid-filled macrophage foam cells [7, 16]. Consequently, macrophage ATP depletion may promote lipid accumulation and expansion of the necrotic core, two key events in progression of atherosclerosis.

In addition to ATP depletion, advanced plaques are also depleted in glucose, especially in the perinecrotic zone. Glucose depletion is most likely due to excessive glycolysis in hypoxic macrophage foam cells in combination with insufficient glucose supply from luminal blood and vasa vasorum [1, 17]. ATP depletion is probably secondary to combined hypoxia and glucose depletion. Combined depletion, but not hypoxia or glucose depletion alone, promotes extensive plaque ATP depletion *ex vivo* [18].

Another consequence of excessive glycolysis is accumulation of lactate. Accumulation of lactate decrease tissue pH and low pH increases intimal lipid retention by increasing affinity of atherogenic lipoproteins for proteoglycans [19]. In addition, low pH decreases macrophage cholesterol efflux [20]. Through these two mechanisms, lactate accumulation in advanced plaques may increase lipid accumulation thus promoting disease progression.

One limitation of our study is that bioluminescence imaging is an in vitro method and analysis of snap frozen tissue is required to reflect the in vivo situation [12]. Human endarterectomies need different times for dissection and during dissection plaque energy metabolites are likely to change. However, an important strength of our study is that the whole endarterectomy specimen was snap frozen in one piece. Each endarterectomy thus contained one large plaque with intermediate histology in the common carotid artery and advanced histology in the internal carotid artery. This allowed direct comparison between intermediate and advanced segments from a single plaque in each patient. Also, the current results in human plaques agree with previous results in rabbit plaques where the atherosclerotic aorta was snap frozen in situ in anaesthetized animals thus closely resembling the in vivo situation [8].

In conclusion, the advanced human plaque is ATP-depleted, deprived of oxygen and glucose, and accumulates lactate. In this respect, the advanced plaque has features of a chronic, non-healing wound [21, 22]. Restoration of ATP levels may decrease necrotic cell death, increase lipid efflux and promote regression of advanced atherosclerotic plaques.
